# Comparing the minimum inhibitory and mutant prevention concentrations of selected antibiotics against animal isolates of *Pasteurella multocida* and *Salmonella typhimurium*

**DOI:** 10.4102/ojvr.v89i1.1955

**Published:** 2022-01-10

**Authors:** Jeanette M. Wentzel, Louise J. Biggs, Moritz van Vuuren

**Affiliations:** 1 Hans Hoheisen Research Station, Faculty of Veterinary Science, University of Pretoria, Pretoria, South Africa; 2 Department of Veterinary Tropical Disease, Faculty of Veterinary Science, University of Pretoria, Pretoria, South Africa; 3 Department of Production Animals, Faculty of Veterinary Science, University of Pretoria, Pretoria, South Africa

**Keywords:** minimum inhibitory concentration, MIC, mutant prevention concentration, MPC, animals, *Salmonella*, *Pasteurella*

## Abstract

Historically, the use of antibiotics was not well regulated in veterinary medicine. The emergence of antibiotic resistance (ABR) in pathogenic bacteria in human and veterinary medicine has driven the need for greater antibiotic stewardship. The preservation of certain antibiotic classes for use exclusively in humans, especially in cases of multidrug resistance, has highlighted the need for veterinarians to reduce its use and redefine dosage regimens of antibiotics to ensure efficacy and guard against the development of ABR pathogens. The minimum inhibitory concentration (MIC), the lowest concentration of an antibiotic drug that will prevent the growth of a bacterium, is recognised as a method to assist in antibiotic dosage determination. Minimum inhibitory concentrations sometimes fail to deal with first-step mutants in bacterial populations; therefore dosing regimens based solely on MIC can lead to the development of ABR. The mutant prevention concentration (MPC) is the minimum inhibitory antibiotic concentration of the most resistant first-step mutant. Mutant prevention concentration determination as a complementary and sometimes preferable alternative to MIC determination for veterinarians when managing bacterial pathogens. The results of this study focused on livestock pathogens and antibiotics used to treat them, which had a MIC value of 0.25 µg/mL for enrofloxacin against all 27 isolates of *Salmonella typhimurium*. The MPC values were 0.50 µg/mL, with the exception of five isolates that had MPC values of 4.00 µg/mL. The MPC test yielded 65.52% (18 isolates) *Salmonella* isolates with florfenicol MICs in the sensitive range, while 11 isolates were in the resistant range. Seventeen isolates (58.62%) of *Pasteurella multocida* had MIC values in the susceptible range and 41.38% (12 isolates) had an intermediate MIC value. Mutant prevention concentration determinations as done in this study is effective for the antibiotic treatment of bacterial infections and minimising the development of resistance. The MPC method can be used to better control to prevent the development of antibiotic drug resistance used in animals.

## Introduction

The growing problem of antibiotic resistance (ABR) is of global concern, with many multidrug resistant bacteria now listed in human medicine. This issue is becoming increasingly relevant in veterinary medicine also, with the risk of resistance genes being transferred between pathogens of humans and animals through various routes and the increasing limitations on antibiotic use in animals, especially food-producing animals.

Various research projects have focused on the role and perspective of the veterinarian in the fight against resistance to antibiotics and its responsible use, including the methods to determine antibiotic selection and dosage (Fortané 2019; Martinez et al. 2014; Trek Diagnostic Systems 2005). It must be borne in mind that resistance to antibiotics while not occurring in every instance of their use can drive resistance when treatments are not fully effective in eliminating bacterial infections (Gebru et al. 2011; Jaganath, Schaaf & Donald 2017). Alternative tests to minimum inhibitory concentration (MIC) determinations and ongoing improvements to determine the most effective dose to treat animals are essential (Martinez et al. 2014). Research has shown that the mutant prevention concentration (MPC) addresses the limitations of MICs in situations such as persistent bacterial infections, where standard dosing is ineffective. This would therefore benefit veterinarians involved in regular antibiotic use situations, such as herd management plans in intensive animal rearing systems. (Firsov et al. 2013).

The MIC testing is currently the most commonly used method in diagnostic laboratories to determine the resistance of bacteria to certain antibiotics. The MIC method determines the lowest concentration (in µg/mL) of an antibiotic that inhibits the growth of a given strain of bacteria and shows the interaction between the drug and the pathogen (Martinez et al. 2014). Minimum inhibitory concentrations can, however, prove ineffective where there is a high rate of mutations in specific organisms such as tuberculosis (Jaganath et al. 2017). With the MPC method, a higher inoculum size (10^8^ coli forming unit (CFU)/mL) is used to block the growth of the least susceptible bacteria present (Coyner 2012).

The MPC values are defined as the antibiotic concentration at which 100% eradication of isolates occurs. The usefulness of MPC lies in the application to calculate the potency of antibiotics along with the comparison to determine the efficacy of different antibiotics against single-step resistant mutants, noting the incidence of resistant mutants (Rodríguez et al. 2004).

When selecting antibiotic type and dose, the use of the MPC method will assist in reducing the bacterial load and will also prevent selective amplification of resistant populations more specifically than MIC determinations (Coyner 2012). The application of MPC values can contribute to a reduction in bacterial resistance, improve therapeutic outcomes and assist responsible use of antibiotics (Gebru et al. 2011). With the increasing importance of resistant bacteria and preserving certain antibiotics for their treatment, veterinarians are under pressure to use antibiotics responsibly, especially in food-producing species. In intensive farming situations with large cohorts of a single species repeatedly being reared in the same environment, careful and targeted antibiotic use is crucial to prevent the emergence and persistence of resistant bacteria. Evidence such as that provided by MPCs can be used by veterinarians to make long-term bacterial disease management plans and help educate farmers regarding the importance of responsible use of antibiotics.

The aim of this study was to illustrate MPC determination as a complementary and sometimes preferable alternative to MIC determination for veterinarians when managing bacterial pathogens. The test results can contribute to the database of MPC values for application in the treatment of livestock.

## Materials and methods

### Sampling and storage

Isolates of *Salmonella typhimurium* and *Pasteurella multocida* from specimens obtained from the Department of Veterinary Tropical Diseases, University of Pretoria, Idexx Laboratories, Disease Control Africa, Stellenbosch Provincial Veterinary Laboratory, Pathcare Veterinary Laboratories and Vetdiagnostix were all confirmed and bio-banked on beads (Cryobank®, Thermo Fischer) at –70 ºC until it could be processed for MIC and MPC tests (Wentzel 2013) (see Table 1).

**TABLE 1:** Demographics on the source for each isolate.

No of samples	Species	Source
*Pasteurella multocida*		
16	Bovine	Trans-tracheal aspirate
9	Bovine	Lung
4	Porcine	Lung
*Salmonella Typhimurium*		
8	Equine	Joint
14	Equine	Faeces
1	Equine	Blood culture
3	Equine	Abscess
1	Equine	Bone

### Biochemical identification of isolates

Isolate confirmation of either *P. multocida* or *S. typhimurium* was done with biochemical assays (Wentzel 2013) (see Table 2) (Songer & Post 2005; Quinn, Carter & Carter 1994) or the Vitek® system (supplied by Biomerieux, Vitek 2XL, France).

**TABLE 2:** Assays for *Salmonella Typhimurium* and *Pasteurella multocida* isolation and confirmation.

Variable	Result
**Test: Salmonella**	
Growth on agar: 1. XLD media	Black colonies on XLD and red colonies on selenite broth
2. McConkey agar	No lactose fermentation
Haemolysis present on blood agar	Negative
Lysine decarboxylase production	Positive
Catalase production	Positive
Glucose & Dulcitol fermentation	Positive
Reaction on triple sugar iron agar	Red slant, yellow butt and black precipitation with precipitation of some H2S
**Test: Pasteurella**	
Growth on selective media	Brain heart broth
Growth on McConkey agar	No Growth
Haemolysis on blood agar	Negative
Oxidase production	Positive with exceptions
Catalase production	Positive
Glucose + sucrose fermentation	Positive
Dulcitol fermentation	Negative
Indole production	Positive with exceptions
Urease production	Negative
L-arabinose fermentation	Negative
D-sorbitol fermentation	Positive
D-Xylose, maltose fermentation	Variable
Nitrate production	Positive

*Source:* Markey, B.K., Leonard, F., Archambault, M., Cullinane, A. & Maguire, D., 2013, Clinical veterinary microbiology, Elsevier, Edinburgh and Songer, J. & Post, K., 2005, *Veterinary microbiology: Bacterial and fungal agents of animal disease*, Elsevier Inv, Philadelphia

XLD, xylose lysine dexycholate; H2S, hydrogen sulfide.

### Antibiotic susceptibility methods

#### Minimum inhibition concentration

The MIC method was done in 96-well microplates (Trek Diagnostic Systems 2005). This quantitative method used breakpoint values to categorise an organism as either a sensitive or a resistant category (Blondeau et al. 2007). The MIC plate preparation was as described in the Clinical Laboratory Standards Institute (CLSI) M31-28 guidelines (Watts et al. 2008). The MIC broth microdilution method was done as per manufacturer instructions of the commercially produced equine (EQUI) and BOPOF (bovine and porcine specific formulary containing FDA approved food animal compounds) Sensititre® MIC plate (Sensititre plates®, Trek Diagnostics, United Kingdom) (Trek Diagnostic Systems 2005). The antibiotic dilution ranges were oxytetracycline/florfenicol at 0.5 µg/mL – 8.0 µg/mL on the BOPOF MIC plate and florfenicol at 0.25 µg/mL – 8.0 µg/mL and 0.25 µg/mL – 2.0 µg/mL enrofloxacin (Trek Diagnostic Systems 2005). Lysed horse blood was added to the BOPOF plates to improve the visual readings of the *P. multocida* reactions. The MIC dilution of *S. typhimurium* was determined using EQUI plates. All samples were tested in duplicate.

#### Mutant prevention concentration

**FIGURE 1:**
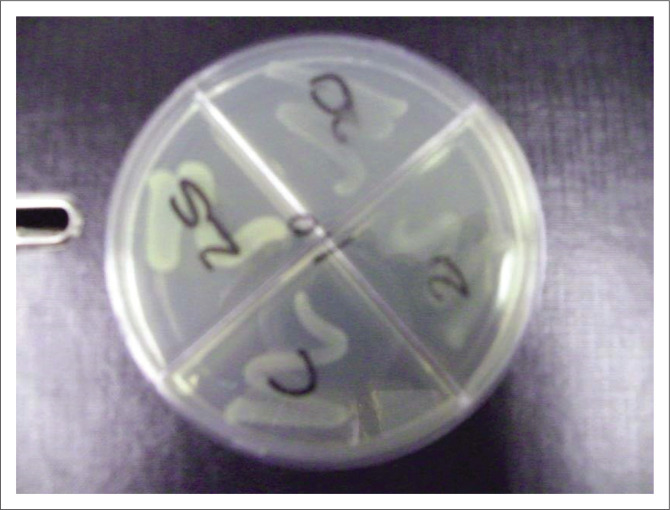
Adapted mutant prevention concentration method plate – With C being the control, the other each section an antibiotic dilution.

The MPC method as described by Blondeau (2009a) was used to determine the MPC values for the *S. typhimurium* isolates (see Figure 1). The MPC detection method utilised agar plates with different concentrations of antibiotic drugs to each plate (i.e. agar dilution method) (Blondeau 2009b), enabling testing one isolate against various antibiotic concentrations in the same time frame. It differs from MIC in that MIC tests are done at 10^5^ CFU/mL bacterial concentrations, whereas the MPC determination is done at a bacterial concentration of 10^9^ CFU/mL (Blondeau 2009a).

Enrofloxacin and oxytetracycline stock solution were prepared in water, while florfenicol was prepared within methanol and stored in a refrigerator (Wentzel 2013). Double serial dilutions of each stock solution were made, starting each dilution at the lowest MIC value obtained (Wentzel 2013). The working concentration of each antibiotic (enrofloxacin-Fluka, 17849, oxytetracycline-Sigma, 04638 and florfenicol-Sigma, F1427) was calculated and used at different concentrations within the Mueller Hinton (MH) agar (Oxoid CM 0337) (Wentzel 2013).

Todd Hewitt broth (Oxoid, CM 0189) was used as enrichment to culture the *P. multocida* isolates replacing MH broth (Quinn et al. 1994). Besides this exception, the method was used as per literature (Blondeau 2009a). The concentration of each isolate was measured against McFarland No. 9 standard (Biomerieux, France) with a spectrophotometer (Densicheck®, Biomerieux) to ensure the density was 10^9^ CFU/mL (Wentzel 2013). Plates were examined after 24 h of incubation at 37 °C for growth with the highest antibiotic concentration with no bacterial growth being the MPC value (µg per mL) (Blondeau et al. 2007). On each plate one quarter was left inoculated with no antibiotic dilution added, this quarter acted as control (see Figure 1).

#### Efficacy determination calculations

Calculated pharmacodynamic/pharmacokinetic values were determined as an indication of bacterial inhibition and effective treatment for each antibiotic (Blondeau et al. 2004). The effective treatment of each antibiotic was determined using the formula area under the curve (AUC)/MIC, with a desired ratio of > 125 for gram-negative and 30–50 for gram-positive organisms for optimal efficacy (Hesje, Tillotson & Blondeau 2007). Each antibiotic’s bacterial inhibition was determined with the formula of *C*_max_ (drug concentration)/MIC and AUC/MIC (Blondeau et al. 2004). These calculations were made using the drug concentrations in the Index of Veterinary Specialities (IVS) (Swan 2005) compared to the data obtained for this study.

### Ethical considerations

Approval to conduct the study was received from the University of Pretoria, Onderstepoort Faculty of Veterinary Science (V063/11).

## Results

The enrofloxacin MIC values of 27 isolates of *S. Typhimurium* were all 0.25 µg/mL, while all MPC values was 0.5 µg/mL, with the exception of five strains being 4.0 µg/mL (Wentzel 2013). The MPC test yielded 18 (65.52%) isolates sensitive to florfenicol, while 11 (34.48%) isolates were resistant to florfenicol. The MIC/MPC ratio of six isolates was either similar or varied by only one dilution (Wentzel 2013).

Seventeen (58.62%) isolates of *P. multocida* had susceptible MIC values and 12 (41.38%) isolates had an intermediate value, while 16 (55.17%) of the isolates yielded a resistant MPC value to oxytetracycline while five isolates had an MIC/MPC ratio of 0 (Wentzel 2013) (see Tables 3 and 4).

**TABLE 3:** Minimum inhibitory concentration and mutant prevention concentration results obtained during current study.

Antibiotic	Organism	No. of samples tested	MIC50	MPC50	MIC50:MPC50	MIC90	MPC90	MIC90:MPC90
			μg/mL	μg/mL	ratio	μg/mL	μg/mL	ratio
Enrofloxacin	*Salmonella* Typhimurium	27	0.25‡	0.5	0.25‡:0.5	0.25‡	4	0.25‡:4
Florfenicol	*Pasteurella*	29	0.50	< 2.0	0.5:< 2.0	2.00	> 32	2:> 32
	*Multocida*	-	-	-	-	-	-	-
Oxytetracycline	*Pasteurella*	29	2.00	16.0	2.0:16	> 8.00	16†	> 8:16†
	*Multocida*	-	-	-	-	-	-	-

MIC, minimum inhibitory concentration; MPC, mutant prevention concentration.

† , Fifty percent to 100% of the isolates yielded an MPC value of > 16.00 μg/mL;

‡ , Hundred percent of the isolates yielded an MIC value of 0.25 μg/mL.

**TABLE 4:** Combined summaries of the pharmacodynamic/pharmacokinetic data for the results obtained for *Pasteurella multocida* and *Salmonella Typhimurium* using reference values from previous research.

Antibiotic	Organism	PD/PK parameter to determine efficacy calculation	Standard measure for efficacy
**AUC/MIC:**			
Enrofloxacin Florfenicol Oxytetracycline	*S. Typhimurium* *P. multocida* *P. multocida*	Not done – Extra-label use 283.56† 56‡	AUC/MIC = 125–250 for optimal efficacy
**Cmax/MIC ratio:**			
Florfenicol Oxytetracycline	P. multocida *P. multocida*	9.38† 2.58‡	Cmax/MIC = 8–12 to minimise resistance

*Source*: Please see the full reference list of the article, Hesje, C.K., Tillotson, G.S. & Blondeau, J.M., 2007, ‘MICs, MPCs and PK/PDs: A match (sometimes) made in hosts’, Expert Review of respiratory medicine 1(1), 7–16. https://doi.org/10.1586/17476348.1.1.7, for more information

*S. Typhimurium, Salmonella Typhimurium; P. multocida, Pasteurella multocida;* AUC/MIC, area under the curve/minimum inhibitory concentration; Cmax/MIC, drug concentration/minimum inhibitory concentration; PD/PK, pharmacodynamics/pharmacokinetics.

† , Concentration of Cmax and AUC, Schering Plough, 2008, for reference values;

‡ , Giguere et al. 2011, for reference values;

§ , Hesje et al., 2007, for reference values.

## Discussion

### General

The clinical breakpoints published in the CLSI guideline refer to the pharmacodynamic and pharmacokinetic attributes of isolates (Boothe 2006). Each clinical breakpoint with respect to the MIC is useful to treat clinical infections but is different from the epidemiological cut-off value that is often lower than the clinical breakpoint. Minimum inhibition concentrations results are divided into three groups being (1) sensitive, (2) intermediate or (3) resistant (Silley, Bywater & Simjee 2006). The susceptibility breakpoint of enrofloxacin for animal pathogens is ≤ 0.5 µg/mL, and the resistance breakpoint is ≥ 4.0 µg/mL (Boothe 2006). None of the MIC values in the current study were resistant. The susceptibility breakpoint of florfenicol for animal pathogens is ≤ 2.0 µg/mL, and the resistance breakpoint is ≥ 8.0 µg/mL (Boothe 2006). None of the MIC values for florfenicol were categorised as resistant during the study. The breakpoint for resistance of oxytetracycline for animal pathogens is ≥ 16 µg/mL, while the susceptibility breakpoint is ≤ 4 µg/mL (Boothe 2006), 12 of the MIC values in the current study were intermediate and remaining susceptible (Wentzel 2013). These breakpoints were used as the reference range in this study. The closer the obtained value to the breakpoint for resistance, the higher the chance of treatment contributing to the development of resistance to the specific antibiotic (Silley et al. 2006).

The MIC and MPC values were used to calculate the pharmacokinetic/pharmacodynamic (PK/PD) parameters. The MPC values in the PD/PK parameter calculation were unknown at the time of the study, and this requires further research (Wentzel 2013). The infection site and dose influence the PK/PD parameters such as AUC (a measure of the total amount of antibiotic drug present over a specific time interval), T > MIC and *C*_max_ (Hesje et al. 2007). Therefore, this study used the values obtained from previously documented studies because it supported the results of the MIC and MPC tests. Previous research indicates that the C_max_/MIC must have a value of 8–12, to be clinically eff ective and to reduce the development of resistance (Hesje et al. 2007). The AUC/MIC should be > 125 to have a positive clinical response and minimise ABR from developing. The AUC/MPC_50_ calculation had a value of ≥ 22, being gram-negative organism, indicating that this treatment can reduce the development of resistance (Hesje et al. 2007).

All of the *S. typhimurium* isolates originated from clinical cases. No official clinical breakpoints exist for enrofloxacin use in animals against *S. typhimurium*; therefore the clinical human breakpoints for *S. typhimurium* and enrofloxacin were used as a guideline in the interpretation of the results. The enrofloxacin clinical reference range is 0.5 µg/mL – 4.0 µg/mL (Watts et al. 2008). The MIC_50_ value of enrofloxacin during this study for *S. typhimurium* was 0.25 µg/mL. This suggests that treating the horses with enrofloxacin was likely adequate when veterinarians use it off label. The few results obtained from this study indicate that enrofloxacin use has not been abused by the equine industry of South Africa to date.

The MPC testing measured the MIC with the most resistant sub population (Gianvecchio et al. 2019) so the *S. typhimurium* isolates with a low MPC_50_ value for enrofloxacin showed the efficacy of the antibiotic against the bacteria. The enrofloxacin use even off label is common in horses; the veterinarians usually treat a horse using similar doses as cattle (Boeckh et al. 2001). The results obtained from the *S. typhimurium* isolates were confirmed with results of previous studies. Studies showed MPC_50_ values with a four-fold increase from the MIC_50_. During this study, the MPC_90_ concentration was 4 µg/mL, thus a 16-fold increase from the MIC_90_. The MPC values above the MPC_50_ will block both susceptible and mutant bacterial growth; alternatively this can be an indication of second-step mutations (Blondeau & Fitch 2019). It is important to know that the MPC will block only the least susceptible bacteria and that it is independent of the mechanism of resistance (Blondeau et al. 2001). Amongst the *S. typhimurium* isolates, there were five strains with MPC values above the MPC_50_ value (Wentzel 2013).

The mutant selection window (MSW) shows the correlation between the MIC_50_ and MPC_50_ values and indicates the effectiveness of the treatment/dosing. This is the concentration where the selective amplification of the organism occurs and where resistant populations can develop (Drlica 2003). Additionally, time-dependent antibiotics that stay within the MSW such as oxytetracycline promote the chances of resistance (Drlica 2003).

Twenty-two (81.48%) of the *S. typhimurium* isolates treated with enrofloxacin yielded results similar to the MIC_50_ and MPC_50_ values (Wentzel 2013).

Isolates included samples from surveillance programmes (44.82%) and clinical cases (55.17%) for the testing of florfenicol and oxytetracycline against *P. multocida,* all isolates had MIC value that was sensitive to florfenicol (Wentzel 2013). The clinical reference range for florfenicol against *P. multocida* infections is 2 µg/mL – 8 µg/mL. Eleven of the isolates had MIC values below the MIC_50_ and eight were suspected (MIC_50_ of 0.5 µg/mL). The MIC of florfenicol for *P. multocida* was within the range when using either the MIC_50_ (0.50 µg/mL) or MIC_90_ (< 2.00 µg/mL) as calculated in this study (Wentzel 2013). Therefore, the treatment of these animals with standard doses of florfenicol suffering from infections with these isolates will be within the therapeutic reference range of the antibiotic. During the current study, the mean MIC concentration of florfenicol for *P. multocida* was slightly higher at 0.50 µg/mL, while the Hörmansdorfer and Bauer (1998) study found the MIC values for *P. multocida* as 0.47 µg/mL for cattle and 0.51 µg/mL for pig strains (Hörmansdorfer & Bauer 1998). Ten *P. multocida* isolates had an MIC_50_ value of 2.00 µg/mL and an MIC_90_ of 4.00 µg/mL for florfenicol, with an MIC_50_ for oxytetracycline of 0.25 µg/mL and the MIC_90_ of 32.00 µg/mL (Sweeney, Brumbaugh & Watts 2008).

The clinical therapeutic reference range of oxytetracycline for *P. multocida* is 4 µg/mL – 16 µg/mL (Blondeau & Fitch 2019). The MIC_50_ value of 2 µg/mL is below the clinical reference range of the antibiotic; 16 isolates (55.17% of samples tested) had MIC values below the clinical breakpoint. Therefore, no resistance was present within these isolates. A single isolate had an MIC value of 4 µg/mL, which is below the reference range, six (20.69% of the samples) of the isolates had an MIC_90_ of 8 µg/mL, while six isolates had an MIC > 8 µg/mL. These results were expected since oxytetracycline is the most commonly used antibiotic drug in cattle in South Africa (Van et al. 2020). These MIC_90_ values are above the clinical breakpoint for resistance, indicating that the treatment of these animals will normally be unsuccessful. Previous research with bovine respiratory disease-causing organisms in cattle had MIC_90_ values of florfenicol and oxytetracycline against *P. multocida* of 0.5 µg/mL and 1.0 µg/mL, respectively (Giguere & Tessman 2011). The MIC_90_ values in the current study were 2 µg/mL and > 8 µg/mL, respectively, both these values are much higher than the reference range as per CLSI guidelines.

The MPC results of the study compared the results obtained with the clinical reference range representing an MPC_50_ of 2 µg/mL and an MPC_90_ of > 32 µg/mL for florfenicol (Blondeau & Fitch 2019). The MPC_50_ results against *P. multocida* were < 2 µg/mL for 16 (62.07% of isolates); these are below the clinical breakpoint for florfenicol. There were 11 isolates of *P. multocida* with MPC values above the clinical reference range of florfenicol. The MPC_90_ concentration represents an alternative to the MIC_50_ values in this study and using higher dosages to exceed the MPC_90_ will theoretically be a more effective treatment regimen to minimise resistance development (Blondeau et al. 2007). Prior to treatment with these higher concentrations (MPC_90_ values), the treatment must be proven safe, as it can be toxic depending on the antibiotic used.

The results for oxytetracycline against *P. multocida* showed an MPC_50_ value of 16 µg/mL for 16 (55.17% of the tested) isolates. Treating animals to reach an MPC_50_ value of 16 µg/mL will be within the clinical reference range of the organism. In this study, both MPC_50_ and MPC_90_ values were 16 µg/mL. None of the *P. multocida* isolates exposed to oxytetracycline had MPC values above the MPC_50_. This creates the need for susceptibility methods such as MPC, which can determine drug concentrations that will kill first-step mutants. The safety of this concentration should be determined first before used for therapy.

During the current study, *P. multocida* had two isolates with similar MIC_50_ and MPC_50_ values for oxytetracycline, and a single isolate had MIC and MPC values within the MSW. None of the *P. multocida* isolates exposed to florfenicol fell between the MIC_50_ and MPC_50_ values. The closer the MIC:MPC ratio is to each other, the higher the suitability of the antibiotic (Zhao & Drlica 2001). The MIC_50_ and MPC_50_ ratios for enrofloxacin against *S. typhimurium* was 0.25:< 0.50 and 0.50:< 2.00 for florfenicol against *P. multocida*, as such the dosages used will be suitable for treatment. The MIC_50_:MPC_50_ ratio of 2:> 16 for oxytetracycline against *P. multocida* as such indicating that treatment at much higher dosages may be needed that might lead to toxicity at the required effective concentration. The MIC_50_:MPC_50_ ratio in this study is similar to the clinical reference range for oxytetracycline. The higher MPC values than the MIC values were expected. The MIC_90_:MPC_90_ ratio for enrofloxacin against *S. typhimirium* was < 0.25:4.00, a 16-fold difference. The MIC_90_:MPC_90_ ratio for florfenicol against *P. multocida* was 2.00:> 32.00, a 16-fold difference, and the MIC_90_:MPC_90_ ratio of > 8:6 for oxytetracycline represents a twofold difference. Enrofloxacin is not registered for use in horses, in South Africa; thus the PK/PD parameters could not be calculated (Swan 2005).

*Pasteurella multocida* isolates responded to florfenicol and were measured with the PD/PK parameters. The AUC/MIC value of 283.56 and an AUC/MIC_90_ value of 70.89 indicated that the treatment will be effective to ensure a positive clinical response; unfortunately the AUC/MIC value of 56 for *P. multocida* isolates exposed to oxytetracycline indicated that treatment would be unsuccessful in these animals (Wentzel 2013).

The *C*_max_/MIC result showed that florfenicol at the MIC_50_ will minimise resistance with a value of 9.38. The oxytetracycline MIC_50_ concentration will not prevent resistance in the *P. multocida* organisms with a value of 2.85 (Wentzel 2013).

Limitations included the initial visual reading of the MIC results; however, after consultation with Trek, adding lysed horse blood to the MH broth before adding the inoculum to the 96-well plates made the reading of the results much easier. The MIC has published known errors with the reading of MIC results, and these include fading end-points (no distinct end-points) or skips (a well with no growth, between wells that have growth) (Trek Diagnostic Systems 2005). These samples were retested in duplicate to confirm the results during this study.

## Conclusion

Distinctions could be made between the MIC_50_, MIC_90_ and the MPC_50_, MPC_90_ for each antibiotic. Applying both methods can be useful for the treatment of highly resistant bacteria and should be investigated further to be more readily available to practitioners. The laboratory interprets the MIC results and provides the information to the practitioner and represents a potentially less toxic and cheaper dosing strategy than MPC. Antibiotic susceptibility testing by means of MIC determinations as done in this study is used for the effective antibiotic treatment of bacterial infections and minimising the development of resistance. The MPC method can be used to better control to prevent the development of antibiotic drug resistance used in animals.
